# Supplementary material for: Genetic population structure of Haemophilus influenzae at local and global scales
Source: Nat Microbiol. 2025 Oct 31;10(12):3136–47. doi: 10.1038/s41564-025-02171-9 (PMC12669038; doi:10.1038/s41564-025-02171-9)
Supplement: Supplementary file 1 — Supplementary Information [file 41564_2025_2171_MOESM1_ESM.pdf]

nature microbiology

Supplementary information

https://doi.org/10.1038/s41564-025-02171-9

Genetic population structure of *Haemophilus influenzae* at local and global scales

In the format provided by the authors and unedited

Supplementary information

Supplementary Table 1. Table of the main PopPUNK clusters, the number of isolates, predominant serotype, cgMLST, and Mismatch 100 cgMLST complex in each cluster. Percentages of the predominant serotype, cgMLST, and 100 mismatch cgMLST complex are provided in parentheses.

Supplementary Table 2. Sequencing QC information for all newly sequenced isolates in this study, including those which failed QC

Supplementary Table 3. Contamination QC information for all the combined collection of the systematic global collection of all publicly available isolates and the newly sequenced Maela collection. QC metrics include the number of genes annotated by Prokka, the number of contigs in the assembled genome, and the distance from the origin of the MDS projection of all pairwise distances as calculated by mash.

Supplementary table 4. Distribution of MDR (resistance against at least four out of nine antibiotic classes) isolates by serotype within pneumonia and non-pneumonia/carriage cases in the Maela host-deduplicated dataset.

Supplementary table 5. Clinical isolate metadata on the Maela cohort, including patient age, collection date and pneumonia status.
