# Supplementary material for: Genetic population structure of Haemophilus influenzae at local and global scales
Source: Nat Microbiol. 2025 Oct 31;10(12):3136–47. doi: 10.1038/s41564-025-02171-9 (PMC12669038; doi:10.1038/s41564-025-02171-9)
Supplement: Supplementary file 2 — Reporting summary [file 41564_2025_2171_MOESM2_ESM.pdf]

nature portfolio

Corresponding author(s): Jukka Corander

Last updated by author(s): Sep 19, 2025

Reporting Summary

Nature Portfolio wishes to improve the reproducibility of the work that we publish. This form provides structure for consistency and transparency in reporting. For further information on Nature Portfolio policies, see our Editorial Policies and the Editorial Policy Checklist.

Statistics

For all statistical analyses, confirm that the following items are present in the figure legend, table legend, main text, or Methods section.

n/a	Confirmed	
☐	☒	The exact sample size (*n*) for each experimental group/condition, given as a discrete number and unit of measurement
☐	☒	A statement on whether measurements were taken from distinct samples or whether the same sample was measured repeatedly
☐	☒	The statistical test(s) used AND whether they are one- or two-sided<br>*Only common tests should be described solely by name; describe more complex techniques in the Methods section.*
☒	☐	A description of all covariates tested
☒	☐	A description of any assumptions or corrections, such as tests of normality and adjustment for multiple comparisons
☐	☒	A full description of the statistical parameters including central tendency (e.g. means) or other basic estimates (e.g. regression coefficient) AND variation (e.g. standard deviation) or associated estimates of uncertainty (e.g. confidence intervals)
☐	☒	For null hypothesis testing, the test statistic (e.g. *F*, *t*, *r*) with confidence intervals, effect sizes, degrees of freedom and *P* value noted<br>*Give P values as exact values whenever suitable.*
☒	☐	For Bayesian analysis, information on the choice of priors and Markov chain Monte Carlo settings
☒	☐	For hierarchical and complex designs, identification of the appropriate level for tests and full reporting of outcomes
☒	☐	Estimates of effect sizes (e.g. Cohen's *d*, Pearson's *r*), indicating how they were calculated

Our web collection on statistics for biologists contains articles on many of the points above.

Software and code

Policy information about availability of computer code

Data collection	Sequence data was produced according to Illumina protocols, and publicly available data was downloaded from the ENA using enadownloader.
Data analysis	No novel algorithms or computational methods were developed during the course of this work.

For manuscripts utilizing custom algorithms or software that are central to the research but not yet described in published literature, software must be made available to editors and reviewers. We strongly encourage code deposition in a community repository (e.g. GitHub). See the Nature Portfolio guidelines for submitting code & software for further information.

Data

Policy information about availability of data

All manuscripts must include a data availability statement. This statement should provide the following information, where applicable:

- Accession codes, unique identifiers, or web links for publicly available datasets
- A description of any restrictions on data availability
- For clinical datasets or third party data, please ensure that the statement adheres to our policy

All sequence data generated as part of this work is available on the ENA/SRA/DDBJ under study accession PRJEB41043. Metadata for both newly sequenced data and the systematic global collection is available as Supplementary Information and on microreact at the links as indicated in figure captions. (https://microreact.org/project/oMm8PFCoG2429JwiDBpdru-maela-h-influenzae and https://microreact.org/project/ioyt4oJRSJgeFGK9KmFyVk-global-h-influenzae-core-tree)

Research involving human participants, their data, or biological material

Policy information about studies with human participants or human data. See also policy information about sex, gender (identity/presentation), and sexual orientation and race, ethnicity and racism.

Reporting on sex and gender	The original Maela cohort study recorded biological sex of infants, as determined by routine physical examination by the attending midwife shortly after birth. The written informed consent process did not explicitly cover reporting of sex in downstream publication. Data on the sex of infants is not reported in the current manuscript.
Reporting on race, ethnicity, or other socially relevant groupings	Maela cohort study participants were from Myanmar and predominantly of Karen ethnicity. Apart from describing the geographic location of Maela camp for displaced persons, the manuscript does not include any race, ethnicity or social group data.
Population characteristics	Maela cohort study participants were displaced persons from Myanmar, residing in Maela camp (northwest Thailand).
Recruitment	Between October 2007 and November 2008, all pregnant women attending the Shoklo Malaria Research Unit (SMRU) antenatal clinic for pregnancy care were invited to consent to their infant's participation in a pneumonia / pneumococcal carriage cohort study. There were no exclusion criteria. The SMRU clinic was the sole provider of antenatal care for Maela camp at that time.
Ethics oversight	Written informed consent was obtained from the participating infants' mothers prior to enrolment into the cohort study. Ethical approval was granted by the ethics committees of the Faculty of Tropical Medicine, Mahidol University, Thailand (MUTM-2009-306) and Oxford University, UK (OXTREC-031-06). The sequencing work on stored isolates described here was approved by the same committees (TMEC-19-043; OxTREC-551-19).

Note that full information on the approval of the study protocol must also be provided in the manuscript.

Field-specific reporting

Please select the one below that is the best fit for your research. If you are not sure, read the appropriate sections before making your selection.

☒ Life sciences ☐ Behavioural & social sciences ☐ Ecological, evolutionary & environmental sciences

For a reference copy of the document with all sections, see nature.com/documents/nr-reporting-summary-flat.pdf

Life sciences study design

All studies must disclose on these points even when the disclosure is negative.

Sample size	As our aim was to study the entire population of H. influenzae in the refugee camp (disease and carriage) we collected as many samples as feasible during routine intervals.
Data exclusions	Sequence data were excluded on the basis of several quality control metrics, including number of sequenced reads, assembly N50, reference genome mapping coverage or presence of contaminant sequences.
Replication	As this is a large whole-genome sequencing study, it is not possible to replicate without repeating the entire study in a novel context.
Randomization	As this is not a comparative study, there are no experimental groups.
Blinding	Investigators analysing the whole-genome sequence data were not involved in the sampling, culturing, or sequencing of any initial biological data.

Reporting for specific materials, systems and methods

We require information from authors about some types of materials, experimental systems and methods used in many studies. Here, indicate whether each material, system or method listed is relevant to your study. If you are not sure if a list item applies to your research, read the appropriate section before selecting a response.

Materials & experimental systems

n/a	Involved in the study
☒	☐ Antibodies
☒	☐ Eukaryotic cell lines
☒	☐ Palaeontology and archaeology
☒	☐ Animals and other organisms
☒	☐ Clinical data
☒	☐ Dual use research of concern
☒	☐ Plants

Methods

n/a	Involved in the study
☒	☐ ChIP-seq
☒	☐ Flow cytometry
☒	☐ MRI-based neuroimaging

Plants

Seed stocks	*Report on the source of all seed stocks or other plant material used. If applicable, state the seed stock centre and catalogue number. If plant specimens were collected from the field, describe the collection location, date and sampling procedures.*
Novel plant genotypes	*Describe the methods by which all novel plant genotypes were produced. This includes those generated by transgenic approaches, gene editing, chemical/radiation-based mutagenesis and hybridization. For transgenic lines, describe the transformation method, the number of independent lines analyzed and the generation upon which experiments were performed. For gene-edited lines, describe the editor used, the endogenous sequence targeted for editing, the targeting guide RNA sequence (if applicable) and how the editor was applied.*
Authentication	*Describe any authentication procedures for each seed stock used or novel genotype generated. Describe any experiments used to assess the effect of a mutation and, where applicable, how potential secondary effects (e.g. second site T-DNA insertions, mosiacism, off-target gene editing) were examined.*
